# Incidence, mortality, and temporal patterns of oropharyngeal cancer in China: a population-based study

**DOI:** 10.1186/s40880-018-0345-5

**Published:** 2018-12-29

**Authors:** Jie Liu, Xu-li Yang, Si-Wei Zhang, Li-Ping Zhu, Wan-Qing Chen

**Affiliations:** 1 Department of Chronic Non-Communicable Diseases Prevention, Jiangxi Province Center for Disease Control and Prevention, Nanchang, Jiangxi 330029 P. R. China; 2 The First Affiliated Hospital of Nanchang University, Nanchang, Jiangxi 330006 P. R. China; 3 Office of Cancer Screening, National Cancer Center/National Clinical Research Center for Cancer/Cancer Hospital, Chinese Academy of Medical Sciences and Peking Union Medical College, Beijing, China

**Keywords:** Oropharyngeal cancer, Incidence, Mortality, Annual percentage change, China

## Abstract

**Background:**

Thus far, the incidence, mortality, and temporal trend data of oropharyngeal cancers (OPC) in China were few. We estimated the incidence, mortality, and temporal patterns of OPC in China during 2008–2012 according to the data from 135 population-based cancer registries to better understand the epidemiological pattern of OPC and to provide more precise information for OPC control in China.

**Methods:**

According to the data of diagnosed OPC reported to 135 cancer registries during 2008–2012, we calculated age-standardized rate of incidence and mortality by 2000 Chinese standard population (ASRIC and ASRMC) and by 1985 Segi’s world standard population (ASRIW and ASRMW) by age, sex, and geographic regions; annual percentage changes of OPC incidence and mortality were calculated using Joinpoint trend analysis.

**Results:**

ASRIW and ASRMW were 2.22/100,000 person-years and 0.94/100,000 person-years, respectively. The incidence and mortality in urban areas were higher than those in rural areas. ASRIC and ASRIW of males were higher than those of females. The overall ASRIC of OPC was significantly increased by 6.2% annually between 2003 and 2006 (*P *= 0.038), but remained stable between 2007 and 2012 (*P *= 0.392). ASRIC and ASRMC of males and in rural areas were significantly increased in the last decade (*P *< 0.05), but the rates of females remained stable during the same period (*P* > 0.05).

**Conclusions:**

Across multiple cancer registries in China, there was an obvious increase in OPC in the recent decade, especially for incidence and mortality of males and in rural areas, whereas the rates of females remained stable. A healthy lifestyle should be advocated and early diagnosis and early treatment of OPC should be enhanced.

**Electronic supplementary material:**

The online version of this article (10.1186/s40880-018-0345-5) contains supplementary material, which is available to authorized users.

## Background

Cancer caused over 8 million deaths and became the second leading cause of death behind cardiovascular disease worldwide [1]. It has become a major public health issue in many countries [2]. The incidence of oral cavity cancers has decreased in recent years in most parts of the world. However, the incidence of oropharyngeal cancers (OPC) has increased over the past decades, predominantly in developed regions of Europe, North America, and parts of East Asia and among males younger than age 60 years [3, 4]. This type of cancer was traditionally considered to be a disease of middle-aged to elderly adults and was mainly observed in males. Nevertheless, increasing numbers of cases have been reported under 45 years of age over the past decades [5, 6]. These observed differences might be related to differences in the etiology of oral cancers at different anatomical sites [3]. Lip, oral cavity, and pharyngeal cancers account for approximately 3.8% of all cancer cases and 3.6% of cancer deaths worldwide; additionally, there was an estimated global age-standardized rate (ASR) of incidence of 1.4/100,000 for OPC (2.3/100,000 for males and 0.5/100,000 for females), and South-central Asia had the highest proportion of new global cases (35.1%) [7]. Literature on the incidence, mortality, and temporal patterns of OPC in China is scarce. Although previous estimates of the national OPC incidence have been reported, they either covered a small proportion of the Chinese population or were focused on specific years [8–10]. Thus far, large-scale epidemiological studies of OPC and information on temporal trends of OPC in China are limited. We estimated the incidence and mortality of OPC in China in 2008–2012 as well as temporal trends for OPC incidence and mortality during 2003–2012 according to the data of 135 population-based cancer registries to better understand OPC epidemiological patterns and to provide more precise scientific information for its control and prevention in China.

## Materials and methods

### Data sources

We quantified 20,618 new diagnoses of OPC and 9635 death cases that were reported to the 135 population-based cancer registries between 1 January 2008 and 31 December 2012 in China. These registries covered a population of 318,623,600 person-years in males and 310,710,310 person-years in females. The 10th revision of the International Statistical Classification of Diseases and Related Health Problems (ICD-10) codes included in this study were C00–C14 (except for C11) [11]. All 135 cancer registries collected data on new cancer cases/deaths from local hospitals, community health centers, vital statistics, or the Civil Administration Bureau [12]. According to the regional division method of the China National Bureau of Statistics, 31 provinces across the country are divided into the eastern, central, and western regions. The 135 cancer registries included 56 in cities (urban areas), covering approximately 382,669,450 person-years, and 79 in counties (rural areas), covering approximately 246,664,460 person-years (Additional file 1: Table S1). Nationwide population data by 5-year sex group and age were provided by statistics or public security census [11]. Individual registries submitted corresponding population databases to the National Central Cancer Registry (NCCR) of China. This information was obtained from the local Public Security/Statistical Bureaus or from calculations on the basis of census data. Thirty-two of these 135 cancer registries, which provided full datasets for new cancer patients diagnosed during 2003–2012 and covered a population of 537,368,837 person-years, were included in incidence/mortality trend analyses (Additional file 1: Table S1). The same standards were used for data collection and report in the two datasets.

#### Statistical indices and methods

 The quality of the dataset from each cancer registry was reviewed and evaluated by NCCR based on the Guidelines for Chinese Cancer Registration and data quality criteria of the International Agency for Research on Cancer/International Association of Cancer Registries (IARC/IACR) [11, 13]. The mortality to incidence (M/I) ratio, the percentage of cancer cases identified with death certification only (DCO%), the proportion of morphologic verification (MV%), and the percentage of uncertified cancer (UB%) were adopted to evaluate the reliability, validity, and completeness of the cancer database [11]. Data from each cancer registry, which was consistently classified as category A or B, were included in our study. Incidence and mortality of OPC were estimated with stratification by gender, area, and age groups. Statistical analysis was conducted using SAS 9.3 version (SAS Institute Inc, Cary, NC, US) and MS EXCEL 2007 (Microsoft, Redmond, WA, USA). IARCcrgTools 2.05, which was issued by IARC/IACR, was adopted for data check and evaluation. Age-standardized rates by standard population of China in 2000 (ASRC) and that of the Segi’s world standard population in 1985 (ASRW) were calculated [8]. Temporal trends of incidence and mortality from 2003 to 2012 (32 registries) were analyzed using Joinpoint Software 4.5 (https://surveillance.cancer.gov/joinpoint/), fitting joinpoint models to the log-transformed data, and the rates were standardized by the world standard population. To reduce the possibility of reporting spurious changes in trends over the period, all models were restricted to a maximum of 2 joinpoints. Trends analysis was expressed as an annual percentage change (APC), and the *Z* test was used to evaluate whether the APC was significantly different from zero. The terms “increase” and “decrease” were adopted when the slope (APC of the trend) was statistically significant (*P *< 0.05).

## Results

### OPC incidence in China

According to the pool of datasets from the 135 cancer registries, 20,618 new cases were diagnosed as OPC between 2008 and 2012 in China: 13,582 in males and 7036 in females. The crude incidence was 3.28/100,000 person-years (4.26/100,000 in males and 2.26/100,000 in females), accounting for 1.16% of overall new diagnosed cancers and ranking the 20th among all cancer sites. Age-standardized rates of incidence by 2000 Chinese standard population (ASRIC) and by 1985 Segi’s world standard population (ASRIW) were 2.27/100,000 person-years and 2.22/100,000 person-years, respectively. The cumulative incidence rate (0–74 years old) of OPC was 0.25%. The incidence in urban areas was higher than that in rural areas. The incidence in males was also higher than that in females. The incidence in the Eastern region was higher than those in the Western region and the Central region (Table 1).

**Table 1:** Oropharyngeal cancer (OPC) incidence in China, 2008–2012

Areas/region	Sex	No. of cases	Incidence (1/100,000)			Cumulative rate (0–74 years old, %)
			Crude	ASRIC	ASRIW	
All	Both sexes	20,618	3.28	2.27	2.22	0.25
	Male	13,582	4.26	3.00	2.96	0.34
	Female	7036	2.26	1.55	1.49	0.17
Urban areas	Both sexes	14,505	3.79	2.54	2.48	0.28
	Male	9568	4.96	3.38	3.32	0.38
	Female	4937	2.60	1.72	1.65	0.19
Rural areas	Both sexes	6113	2.48	1.82	1.77	0.21
	Male	4014	3.20	2.37	2.34	0.28
	Female	2099	1.73	1.27	1.22	0.14
Eastern regions	Both sexes	15,433	3.49	2.31	2.25	0.26
	Male	10,171	4.56	3.07	3.02	0.35
	Female	5262	2.40	1.56	1.50	0.17
Central regions	Both sexes	3627	2.75	2.14	2.09	0.24
	Male	2399	3.55	2.81	2.77	0.32
	Female	1228	1.91	1.48	1.43	0.16
Western regions	Both sexes	1558	2.86	2.25	2.21	0.25
	Male	1012	3.64	2.94	2.91	0.33
	Female	546	2.04	1.58	1.53	0.17

Cumulative rates, the rates for patients aged 0–74 years, are a special form of standardized rates in which equal weights are given for all 5-year age groups up to a defined upper age limit, which in this case is 75 years. For each year included in the present study, age-specific rates were computed and multiplied by the length of the age group in years (5 years). These were then summed to derive the cumulative incidence rates

*ASRIC* age-standardized rate of incidence by 2000 Chinese standard population, *ASRIW* age-standardized rate of incidence by 1985 Segi’s world standard population

### OPC mortality in China

There were 9335 death cases of OPC in China between 2008 and 2012, which included 6575 males and 2760 females. The crude mortality was 1.48/100,000 person-years, accounting for 0.83% of overall cancer deaths, and was ranked the 18th among all cancer sites. Age-standardized rates of mortality by 2000 Chinese standard population (ASRMC) and by 1985 Segi’s world standard population (ASRMW) were 0.95/100,000 and 0.94/100,000, respectively. The cumulative death rate (0–74 years old) was 0.10%. The mortality of OPC was higher in males than in females. The mortality in urban areas was higher than that of rural areas. The mortality in females was lower than that in males. The ASRMC and ASRMW in the Western region were higher than those in the Eastern and Central regions (Table 2).

**Table 2:** Oropharyngeal cancer mortality in China, 2008–2012

Areas/region	Sex	No. of cases	Mortality (1/100,000)			Cumulative rate (0–74 years old, %)
			Crude	ASRMC	ASRMW	
All	Both sexes	9335	1.48	0.95	0.94	0.10
	Male	6575	2.06	1.39	1.39	0.15
	Female	2760	0.89	0.53	0.52	0.05
Urban areas	Both sexes	6420	1.68	1.03	1.02	0.11
	Male	4547	2.36	1.52	1.52	0.17
	Female	1873	0.99	0.55	0.54	0.05
Rural areas	Both sexes	2915	1.18	0.82	0.81	0.09
	Male	2028	1.62	1.16	1.15	0.13
	Female	887	0.73	0.49	0.48	0.05
Eastern regions	Both sexes	7051	1.59	0.96	0.95	0.10
	Male	4930	2.21	1.40	1.40	0.15
	Female	2121	0.97	0.53	0.52	0.05
Central regions	Both sexes	1599	1.21	0.91	0.90	0.10
	Male	1155	1.71	1.33	1.33	0.15
	Female	444	0.69	0.49	0.49	0.06
Western regions	Both sexes	685	1.26	0.98	0.97	0.11
	Male	490	1.76	1.42	1.43	0.16
	Female	195	0.73	0.55	0.53	0.06

*ASRMC* age-standardized rate of mortality by 2000 Chinese standard population, *ASRMW* age-standardized rate of mortality by 1985 Segi’s world standard population

### Age-specific incidence and mortality of OPC

**Fig. 1:**
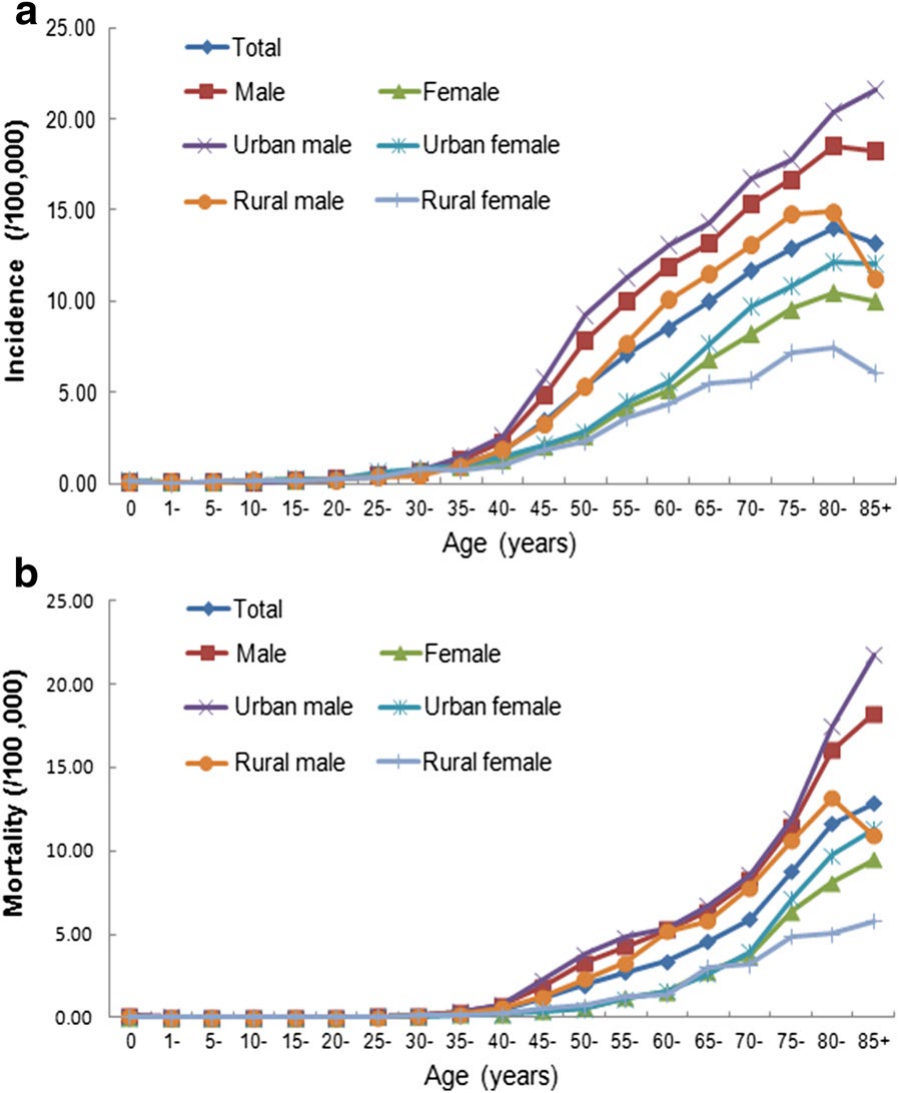
Age-specific incidence and mortality of oropharyngeal cancer (OPC) in China, 2008–2012. **a** The age-specific incidence increased remarkably from age 40–45, peaked at age 80–84 for both males and females, and decreased thereafter. The incidence in males was higher than that in females; the incidence in urban areas was higher than that in rural areas. **b** The age-specific mortality increased remarkably from age 40–45, peaked at age 80–84 for males and at age 85 and above for females. The mortality in males was higher than that in females; the mortality in urban areas was higher than that in rural areas

The incidence of OPC was relatively low before age 30, and then began to rise slowly; it increased dramatically after age 40, peaked at age 80, and then slightly decreased after age 85 (Fig. 1a). The age-specific incidence was higher in urban areas than in rural areas in almost all the age-groups (except for the 0–10 years age group). The incidence in males was higher in urban areas than in rural areas after age 15, whereas the incidence in females was higher in urban areas than in rural areas in every age group after age 35.

The mortality of OPC was relatively low before age 40, then began to increase, and reached a peak after age 85 (Fig. 1b). The mortality of OPC in rural areas was the highest at age 80–84. The age-specific mortality in males was higher in urban areas than in rural areas after age 25, whereas the mortality in females was higher in urban areas than in rural areas after age 70. The mortality in urban areas was higher than that in rural areas after age 35.

### Time trend analysis

**Fig. 2:**
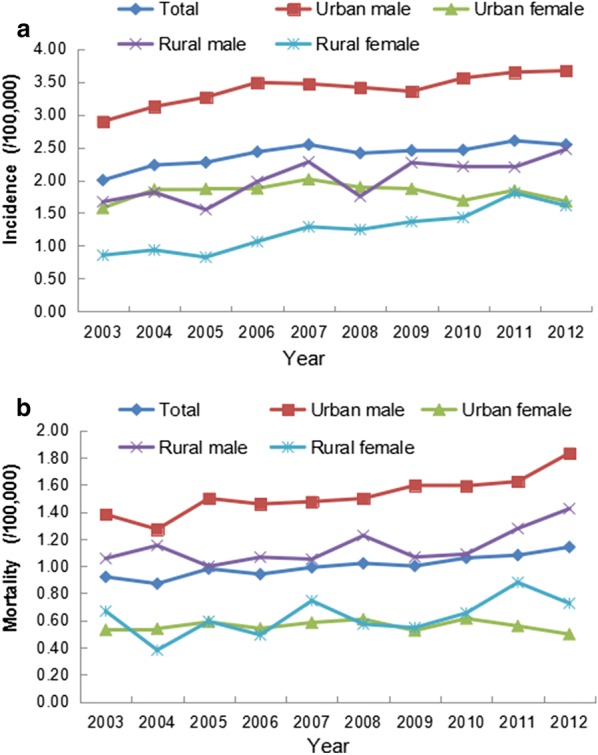
Trends of ASRIC and ASRMC of OPC in China, 2003–2012. **a** The ASRIC increased during 2003–2006, remained stable during 2007–2012. The OPC incidence increased in males, especially in rural areas. **b** The ASRMC was stable over the study period (2003–2012) for females, whereas significant upward trends were observed for males. There was an increase in mortality over the time period both in urban and rural areas

The ASRIC of OPC increased during 2003–2006 (annual percentage change [APC] = 6.2%, 95% confidence interval [CI] = 0.5% to 12.3%) and remained stable during 2007–2012 (APC = 0.7%, 95% CI = − 1.2% to 2.6%). Overall, the OPC incidence increased for males (APC = 2.5%, 95% CI = 1.5% to 3.5%) but not for females (APC = 5.5%, 95% CI = − 0.9% to 12.3%). A marginally significant temporal trend of ASRIC was observed in urban areas (APC = 8.5%, 95% CI = 0.0 to 17.6%, *P *= 0.049). These data reveal a significant increase of OPC in China in the recent decade, especially for males in rural areas (Tables 3, 4, Fig. 2a).

**Table 3:** Trends of incidence and mortality of OPC in China, 2003–2012

Item	PC (%)	Trend 1^#^					Trend 2^#^				
		APC (%)	95% CI (%)	Period	*t*	*P*	APC (%)	95% CI (%)	Period	*t*	*P*
Incidence											
Total	27.0	6.2	0.5 to 12.3	2003–2006	2.802	0.038	0.7	− 1.2 to 2.6	2007–2012	0.937	0.392
Urban areas	20.4	8.5	0.0 to 17.6	2003–2005	2.582	0.049	0.3	− 0.8 to 1.4	2006–2012	0.633	0.554
Rural areas	61.1	6.0	3.8 to 8.2	2003–2012	6.333	< 0.001	–	–	–	–	–
Males	30.7	2.5	1.5 to 3.5	2003–2012	5.951	< 0.001	–	–	–	–	–
Females	17.7	5.5	− 0.9 to 12.3	2003–2007	2.181	0.081	− 1.6	− 5.9 to 2.9	2008–2012	− 0.932	0.394
Mortality											
Total	23.9	2.5	1.7 to 3.3	2003–2012	7.165	< 0.001	–	–	–	–	–
Urban areas	22.3	2.3	1.4 to 3.1	2003–2012	6.037	< 0.001	–	–	–	–	–
Rural areas	25.9	3.2	0.9 to 5.6	2003–2012	3.275	0.011	–	–	–	–	–
Males	33.6	2.9	2.0 to 3.9	2003–2012	7.156	< 0.001	–	–	–	–	–
Females	− 3.6	0.8	− 1.1 to 2.7	2003–2012	0.945	0.372	–	–	–	–	–

*PC* percentage change, *APC* annual percentage change, *CI* confidence interval

^#^The program started with the minimum number of joinpoint (e.g., 0 joinpoint, which is a straight line) and tested whether more joinpoints are statistically significant and must be added to the model (up to that maximum number), enabling the user to test whether an apparent change in trend is statistically significant. The significance was tested using a Monte Carlo Permutation method. To reduce the possibility of reporting spurious changes in trends over the period, all models were restricted to a maximum of 2 joinpoints

**Table 4:** Temporal trends of ASRIC of OPC in China, 2003–2012

Year	ASRIC (1/100,000)								
	Total			Urban areas			Rural areas		
	Both sexes	Males	Females	Both sexes	Male	Female	Both sexes	Male	Female
2003	2.00	2.64	1.41	2.21	2.89	1.58	1.26	1.67	0.86
2004	2.23	2.85	1.66	2.47	3.12	1.86	1.35	1.82	0.94
2005	2.27	2.93	1.65	2.55	3.26	1.87	1.18	1.55	0.83
2006	2.43	3.20	1.71	2.66	3.49	1.87	1.50	1.98	1.06
2007	2.54	3.24	1.87	2.73	3.47	2.01	1.77	2.28	1.29
2008	2.41	3.08	1.75	2.64	3.41	1.89	1.50	1.75	1.25
2009	2.45	3.14	1.77	2.60	3.35	1.87	1.81	2.27	1.37
2010	2.46	3.31	1.64	2.61	3.56	1.69	1.82	2.21	1.43
2011	2.60	3.36	1.84	2.74	3.64	1.85	1.99	2.20	1.81
2012	2.54	3.45	1.66	2.66	3.67	1.68	2.03	2.47	1.61

*ASRIC* age-standardized rate of incidence by 2000 Chinese standard population

The ASRMC was stable over the study period (2003–2012) for females (APC = 0.8%, 95% CI = − 1.1% to 2.7%), whereas significant upward trends were observed for males (APC = 2.9%, 95% CI = 2.0% to 3.9%). There was an increase in ASRMC over the time period in both urban areas (APC = 2.3%, 95% CI = 1.4% to 3.1%) and rural areas (APC = 3.2%, 95% CI = 0.9% to 5.6%) (Tables 3, 5, Fig. 2).

**Table 5:** Temporal trends of ASRMC of OPC in China, 2003–2012

Year	ASRMC (1/100,000)								
	Total			Urban areas			Rural areas		
	Both sexes	Males	Females	Both sexes	Males	Females	Both sexes	Males	Females
2003	0.92	1.31	0.56	0.94	1.38	0.53	0.85	1.06	0.67
2004	0.87	1.25	0.51	0.89	1.27	0.54	0.75	1.15	0.38
2005	0.98	1.40	0.59	1.03	1.50	0.59	0.78	1.00	0.59
2006	0.94	1.38	0.53	0.99	1.46	0.54	0.76	1.07	0.49
2007	0.99	1.40	0.61	1.02	1.47	0.58	0.89	1.05	0.74
2008	1.02	1.45	0.60	1.04	1.50	0.61	0.89	1.22	0.57
2009	1.00	1.49	0.53	1.05	1.59	0.52	0.80	1.07	0.55
2010	1.06	1.50	0.62	1.10	1.59	0.61	0.87	1.09	0.65
2011	1.08	1.56	0.62	1.08	1.62	0.56	1.07	1.28	0.88
2012	1.14	1.75	0.54	1.15	1.83	0.50	1.07	1.42	0.73

*ASRMC* age-standardized rate of mortality by 2000 Chinese standard population

## Discussion

The current analysis included 20,618 new diagnoses of OPC and 9335 death cases. Our data showed that the ASRIW of OPC was 2.22/100,000 and the ASRMW was 0.94/100,000 in China. Additionally, the overall ASRIC of OPC significantly increased by 6.2% annually during 2003–2006 (*P *< 0.05), but remained stable during 2007–2012. The ASRIC and ASRMC for males and in rural areas significantly increased in the recent decade (*P *< 0.05); the incidence significantly increased by 30.7% in males (*P *< 0.01) and by 61.1% in rural areas (*P *< 0.01). Nevertheless, ASRIC and ASRMC remained stable in females during those periods. The ASRIW of OPC in China was slightly above the average level of Colombia, Costa Rica, and Ecuador [3], but lower than the United States of America (15.6/100,000 for males and 6.1/100,000 for females) [14]. In contrast, the incidence of OPC in males is high in North France with a rate of 42.3/100,000 in males in the Somme and Bas Rhin regions [14].

Tobacco smoking, alcohol consumption, and human papillomavirus types 16 and 18 (HPV16/18) infections were identified as the major risk factors for OPC, with tobacco smoking and alcohol consumption having synergistic effects [15–17]. Tobacco use is a mutagenic factor that increases the risk of oral cavity and pharyngeal cancers [18]. China constitutes approximately 40% of the world’s total tobacco use, predominantly in males, making China the largest tobacco consumer worldwide [19]. Additionally, a large increase in consumption was observed in urban rather than rural areas over the past three decades [19]. It might partly explain the reason why the incidence of OPC was higher in urban areas than in rural areas. In addition, although the mechanisms for alcohol-related carcinogenesis were not completely clarified, the IARC listed both alcoholic beverages and acetaldehyde, including its major metabolites, as human carcinogens. Alcohol drinking is an established risk factor for head and neck cancer, and this relationship was even stronger among cancers of the oropharynx and hypopharynx [20]. Li et al. [21] reported that the proportions of current drinkers among Chinese males, females, and the whole population were 55.6%, 15.0%, and 35.7%, respectively. Additionally, among current drinkers, the proportions of excessive, frequent, and binge drinkers were 59.1%, 26.0%, 53.7% in urban areas and 64.7%, 26.4%, 59.3% in rural areas, respectively. Furthermore, a significant association between heavy alcohol drinking and malnutrition was observed, which may have led to reduced intake of vegetables, fruits, and some other foods with cancer-preventive effects [22]. Additionally, the local cytotoxic activity of ethanol might explain the synergistic effect of tobacco and alcohol consumption on the risk of OPC. The cytotoxic effect of ethanol on the cells lining these tissues activated the division of stem cells located in deeper layers to replace dead cells. By activating their division, alcohol leaves the DNA of stem cells highly exposed to the DNA-damaging activity of tobacco carcinogens [23].

In head and neck squamous cell carcinoma, HPV has been regarded as an important pathogen, in addition to alcohol and tobacco consumption [24]. An increase in the incidence of oropharyngeal squamous cell carcinoma (OPSCC) was observed in some countries [3, 4], and this has been attributed to HPV infection [25]. Currently, the natural history of oral HPV infection has not been fully studied [26]. Nevertheless, some progress has been made in recent decades. It was reported that HPV DNA was detected in approximately 19%–75% of OPSCCs worldwide, 85%–95% of which belong to the HPV-16 type [27, 28]. A recent study performed in China also showed that HPV infection was detected in 16.7% of OPSCC specimens [27], which was similar to the detection in 18%–36% of OPSCCs reported in studies from developed countries [28]. The development of HPV-associated OPSCC was found to be associated with high-risk sexual behaviours, including an increased number of sexual partners/oral sex partners, and homosexual intercourse [3]. In addition, a significantly higher prevalence of oral HPV infection was found in subjects who had performed oral sex than in subjects who had not, consistent with previous studies that showed that oral sex might be a risk factor for oral HPV infection [29]. Using data from cancer registries and data of HPV prevalence, recent studies in Australia [30], the United States [31], and Sweden [32] have indicated that changes in sexual behaviours among recent birth cohorts have led to increased oral HPV exposure and to an increased incidence of OPC. Nevertheless, the natural history of oral HPV infection is still largely unknown, and ongoing research is underway to explore why males are more susceptible to acquisition and persistent HPV infection than females [33].

The present study demonstrated temporal trends of OPC incidence and mortality according to the ICD-10 classification. Joinpoint analysis provided a clear picture of temporal patterns in different segments of time. As such, we were able to show the significant changes in trends that have occurred during the study period. Using the APC model, we detected significant differences in incidence and mortality trends of OPC between males and females. Furthermore, OPC incidence significantly increased in rural and urban areas among males. The present study confirmed the increases in incidence and mortality of OPC in the last decade, especially for males in rural areas. Nevertheless, the ASRIC and ASRMC were stable over the study period for females, which may be because of an increased awareness of the need of sunscreen or low smoking rates in females [19]. Our results were consistent with previous studies on trends of OPC in other countries [16, 34, 35].

Our observations of increasing OPC incidence in males and in rural areas help us better understand its epidemiological situation in China and provide more precise scientific information for its control and prevention. The main strength of our study is the population-based design with a large sample size of OPC patients in China. Furthermore, we included 32 cancer registries that had data of sufficient quality over the 10-year period (2003–2012) for inclusion in incidence and mortality trend analyses. Additionally, our epidemiologic study was nationwide and covered a period of 5 years.

Nevertheless, there were limitations that should be addressed. First, the information submitted by the cancer registries was incomplete because the private behaviours such as oral/vaginal sexual behaviours were not known. Second, it did not contain information of other risk factors, such as individual tobacco and alcohol consumption. Therefore, the effect of these factors on temporal trends could only be assessed. Finally, the small number of cases in some subgroups (i.e., small number of death cases in the Western region) may preclude further analysis by age in females.

## Conclusions

We analyzed temporal trends of incidence and mortality of OPC by sex and geographic regions using a large sample from population-based cancer registries in China. The results confirmed an increase in OPC incidence and mortality in the recent decade, especially for males in rural areas. However, the age-standardized rates were stable over the study period for females. The implementation of public health measures such as screening programms and risk factor control should be adopted for reducing the incidence in China. Studies with larger sample sizes are needed to further explore the roles of HPV infection, tobacco and alcohol consumption, and sexual behaviours in the development of OPC.

## Additional file

**Additional file 1: Table S1.** List of 135 cancer registries which provided full datasets for new cancer patients diagnosed during 2008–2012.

## Abbreviations

ASR: age-standardized rate
OPC: oropharyngeal cancer
ASRIC: age-standardized rate of incidence by 2000 Chinese standard population
ASRIW: age-standardized rate of incidence by 1985 Segi’s world standard population
ASRMC: age-standardized rate of mortality by 2000 Chinese standard population
ASRMW: age-standardized rate of mortality by 1985 Segi’s world standard population
ICD-10: the 10th revision of the International Statistical Classification of Diseases and Related Health Problems
NCCR: National Central Cancer Registry of China
IARC/IACR: International Agency for Research on Cancer/International Association of Cancer Registries
M/I: mortality to incidence
DCO%: the percentage of cancer cases identified with death certification only
MV%: the proportion of morphologic verification
UB%: the percentage of uncertified cancer
APC: annual percentage change
HPV: human papillomavirus
OPSCC: oropharyngeal squamous cell carcinoma
