# Correction to: Incidence, mortality, and temporal patterns of oropharyngeal cancer in China: a population-based study

**DOI:** 10.1186/s40880-019-0352-1

**Published:** 2019-02-26

**Authors:** Jie Liu, Xu-li Yang, Si-Wei Zhang, Li-Ping Zhu, Wan-Qing Chen

**Affiliations:** 1 Department of Chronic Non-Communicable Diseases Prevention, Jiangxi Province Center for Disease Control and Prevention, Nanchang, Jiangxi 330029 P. R. China; 2 The First Affiliated Hospital of Nanchang University, Nanchang, Jiangxi 330006 P. R. China; 3 Office of Cancer Screening, National Cancer Center/National Clinical Research Center for Cancer/Cancer Hospital, Chinese Academy of Medical Sciences and Peking Union Medical College, Beijing, China

## Correction to: Cancer Commun (2018) 38:75 10.1186/s40880-018-0345-5

In the original publication of this article [1], the number of death cases in ‘Materials and methods’ section is incorrect.

The original sentence is ‘We quantified 20,618 new diagnoses of OPC and 9635 death cases that were reported to the 135 population-based cancer registries between 1 January 2008 and 31 December 2012 in China’. The correct number of death cases is 9335.
