# Supplementary material for: Incidence, mortality, and temporal patterns of oropharyngeal cancer in China: a population-based study
Source: Cancer Commun (Lond). 2018 Dec 29;38:75. doi: 10.1186/s40880-018-0345-5 (PMC6311068; doi:10.1186/s40880-018-0345-5)
Supplement: Supplementary file 1 — Additional file 1: Table S1. List of 135 cancer registries which provided full datasets for new cancer patients diagnosed during 2008–2012. [file 40880_2018_345_MOESM1_ESM.docx]

Table S1 List of 135 cancer registries which provided full datasets for new cancer patients diagnosed during 2008-2012

| **Region** | **Province/municipality** | **City/county** | **Registry number** | **Code (1 = city; 2 = county)** |
| --- | --- | --- | --- | --- |
| Eastern | Beijing | Beijing City | 110100^*^ | 1 |
|  |  | Suburban county, Beijing | 110200 | 2 |
|  | Tianjing | Tianjing City | 120100 | 1 |
|  | Hebei | Huangzan County | 130129 | 2 |
|  |  | Qianxi County | 130227 | 2 |
|  |  | Qinghuangdao City | 130301 | 1 |
|  |  | Shexian County | 130426^*^ | 2 |
|  |  | Cixian | 130427^*^ | 2 |
|  |  | Wuan County | 130481 | 2 |
|  |  | Baoding City | 130600 | 1 |
|  | Liaoning | Shenyang City | 210101^*^ | 1 |
|  |  | Dalian City | 210200^*^ | 1 |
|  |  | Zhuanghe County | 210283 | 2 |
|  |  | Anshan City | 210300^*^ | 1 |
|  |  | Benxi City | 210501^*^ | 1 |
|  |  | Dandong City | 210601 | 1 |
|  |  | Donggang County | 210681 | 2 |
|  |  | Jianping County | 211322 | 2 |
|  | Shanghai | Shanghai City | 310100^*^ | 1 |
|  | Jiangsu | Wuxi City | 320200 | 1 |
|  |  | Changzhou City | 320400 | 1 |
|  |  | Jintan County | 320482^*^ | 2 |
|  |  | Suzhou City | 320501 | 1 |
|  |  | Haian County | 320621 | 2 |
|  |  | Qidong County | 320681^*^ | 2 |
|  |  | Haimen County | 320684^*^ | 2 |
|  |  | Lianyungang City | 320701 | 1 |
|  |  | Ganyu County | 320721 | 2 |
|  |  | Donghai County | 320722 | 2 |
|  |  | Guanyun County | 320723 | 2 |
|  |  | Huaian District, Huaian City | 320803^*^ | 1 |
|  |  | Huaiyin District, Huaian City | 320804 | 1 |
|  |  | Lianshui County | 320826 | 2 |
|  |  | Hongze County | 320829 | 2 |
|  |  | Xuyi County | 320830 | 2 |
|  |  | Jinhu County | 320831 | 2 |
|  |  | Yancheng City | 320900 | 1 |
|  |  | Binhai County | 320922 | 2 |
|  |  | Sheyang County | 320924 | 2 |
|  |  | Jianhu County | 320925^*^ | 2 |
|  |  | Dafeng County | 320982^*^ | 2 |
|  |  | Yangzhong County | 321182^*^ | 2 |
|  |  | Taixing County | 321283 | 2 |
|  | Shandong | Tengzhou | 370481 | 2 |
|  |  | Zhaoyuan County | 370685 | 2 |
|  |  | Linju County | 370724^*^ | 2 |
|  |  | Wenshang County | 370830 | 2 |
|  |  | Zoucheng | 370883 | 2 |
|  |  | Ningyang | 370921 | 2 |
|  |  | Feicheng | 370983^*^ | 2 |
|  |  | Rushan County | 371083 | 2 |
|  |  | Junan County | 371327 | 2 |
|  | Zhejiang | Hangzhou City | 330101^*^ | 1 |
|  |  | Cixi | 330282 | 2 |
|  |  | Jiaxing City | 330401^*^ | 1 |
|  |  | Jiashan County | 330421^*^ | 2 |
|  |  | Haining County | 330481^*^ | 2 |
|  |  | Shangyu District, Shaoxing County | 330682 | 2 |
|  |  | Xianju County | 331024 | 2 |
|  | Fujian | Changle | 350182^*^ | 2 |
|  |  | Xiamen City | 350201 | 1 |
|  |  | Tongan District, Xiamen City | 350212 | 1 |
|  |  | Hanjiang District, Putian City | 350303 | 1 |
|  | Guangdong | Guangzhou City | 440101^*^ | 1 |
|  |  | Suburb of Guangzhou | 440109 | 2 |
|  |  | Shenzhen City | 440301 | 1 |
|  |  | Zhuhai City | 440400 | 1 |
|  |  | Urban district, Jiangmen | 440701 | 1 |
|  |  | Sihui | 441284^*^ | 2 |
|  |  | Zhongshan City | 442000^*^ | 1 |
| Central | Shanxi | Yangquan City | 140300 | 1 |
|  |  | Yangcheng County | 140522^*^ | 2 |
|  | Jilin | Dehui County | 220183 | 2 |
|  |  | Tonghua City | 220500 | 1 |
|  |  | Yanji County | 222401 | 2 |
|  | Heilongjiang | Daoli District, Harbin City | 230102 | 1 |
|  |  | Nangang District, Harbin City | 230103^*^ | 1 |
|  | Anhui | Hefei City | 340100 | 1 |
|  |  | Feixi County | 340123 | 2 |
|  |  | Maanshan City | 340501^*^ | 1 |
|  |  | Tongling City | 340700 | 1 |
|  |  | Yingdong District, Fuyang City | 341203 | 1 |
|  |  | Lingbi County | 341323 | 2 |
|  |  | Shouxian County | 341521 | 2 |
|  |  | Jinxian County | 341823 | 2 |
|  | Jiangxi | Wuning County | 360423 | 2 |
|  |  | Zhanggong District, Ganzhou City | 360702 | 1 |
|  | Henan | Luoyang | 410300 | 1 |
|  |  | Yanshi | 410381 | 2 |
|  |  | Lushan County | 410423 | 2 |
|  |  | Linzhou | 410581^*^ | 2 |
|  |  | huixian County | 410782 | 2 |
|  |  | Yuzhou | 411081 | 2 |
|  |  | yuanhui District, Luohe | 411102 | 1 |
|  |  | Yancheng District, Luohe | 411103 | 1 |
|  |  | Zhaoling District, Luohe | 411104 | 1 |
|  |  | Neixiang | 411325 | 2 |
|  |  | Yucheng | 411425 | 2 |
|  |  | Luoshan | 411521 | 2 |
|  |  | Shenqiu County | 411624 | 2 |
|  |  | Dancheng | 411625 | 2 |
|  |  | Xiping County | 411721 | 2 |
|  |  | Jiyuan City | 419001 | 1 |
|  | Hubei | Wuhan City | 420101^*^ | 1 |
|  |  | Wufeng Tujia Autonomous County | 420529 | 2 |
|  |  | Yunmeng County | 420923 | 2 |
|  |  | Gongan County | 421022 | 2 |
|  | Hunan | Shifeng District, Zhuzhou City | 430204 | 1 |
|  |  | Hengdong County | 430424 | 2 |
|  |  | Yueyanglou District, Changsha City | 430602 | 1 |
|  |  | Cili County | 430821 | 2 |
|  |  | Zixing County | 431081 | 2 |
|  |  | Mayang County | 431226 | 2 |
| Western | Inner Mongolia | Cifeng City | 150400 | 1 |
|  |  | Kailu County | 150523 | 2 |
|  |  | Yakeshi City | 150782 | 1 |
|  | Guangxi | Liuzhou City | 450201 | 1 |
|  |  | Guilin City | 450300 | 1 |
|  |  | Hepu County | 450521 | 2 |
|  |  | Fusui County | 451421^*^ | 2 |
|  | Chongqing | Wanzhou District, Chongqing City | 500101 | 2 |
|  |  | Yuzhong District, Chongqing City | 500103 | 1 |
|  |  | jiulongpo district, Chongqing City | 500107 | 1 |
|  | Sichuan | Qingyang District, Chengdu City | 510105 | 1 |
|  |  | Pengzhou, Chengdu | 510182 | 2 |
|  |  | Yanting County | 510723^*^ | 2 |
|  | Yunnan | Hongta District, Yuxi City | 530402 | 1 |
|  |  | Gejiu | 532501 | 2 |
|  | Gansu | Jingtai County | 620423 | 2 |
|  |  | Liangzhou District, Wuwei City | 620602 | 1 |
|  |  | Ganzhou District, Zhangye City | 620702 | 1 |
|  |  | Lintan County | 623021 | 2 |
|  | Qinghai | Xining City | 630100 | 1 |
|  | Ningxia | Yinchuan City | 640100 | 1 |
|  | Xinjiang | Xinyuan County | 654025 | 2 |

*These registries provided full datasets for new cancer patients diagnosed during 2003-2012.
